# Near‐Whole‐Genome Sequencing of Peste Des Petits Ruminants Virus Lineage IV From the Savannah District, Northern Côte d’Ivoire in 2023

**DOI:** 10.1155/vmi/6654668

**Published:** 2026-09-02

**Authors:** Konan Ange-Sylvestre Goli, Jean Nepomuscene Hakizimana, Augustino Alfred Chengula, Kiffopan Benjamin M’Bari, Charlie Franck Amoia, Melvyn Quan, Mariam Makange, Peter N. Thompson, Gerald Misinzo

**Affiliations:** ^1^ Department of Microbiology, Parasitology and Biotechnology, College of Veterinary Medicine and Biomedical Sciences, Sokoine University of Agriculture, P.O. Box 3019, Morogoro 67125, Tanzania, suanet.ac.tz; ^2^ Department of Veterinary Tropical Diseases, Faculty of Veterinary Science, University of Pretoria, Private Bag X04, Onderstepoort 0110, South Africa, up.ac.za; ^3^ SACIDS Africa Centre of Excellence for Infectious Diseases, SACIDS Foundation for One Health, Sokoine University of Agriculture, P. O. Box 3297, Morogoro 67152, Tanzania, suanet.ac.tz; ^4^ OR Tambo Africa Research Chair for Viral Epidemics, SACIDS Foundation for One Health, Sokoine University of Agriculture, P.O. Box 3297, Morogoro 67125, Tanzania, suanet.ac.tz; ^5^ Animal Biology, Agropastoral Management Institute, Peléforo Gon Coulibaly University, BP 1328, Korhogo, Côte d’Ivoire; ^6^ Department of Biology-Geology, Faculty of Science and Technology, Alassane Ouattara University, BP V 18, Bouaké 01, Côte d’Ivoire, univ-ao.edu.ci; ^7^ Department of Production Animal Studies, Faculty of Veterinary Science, University of Pretoria, Private Bag X04, Onderstepoort 0110, South Africa, up.ac.za

**Keywords:** goat, lineage IV, nanopore sequencing, PPRV, sheep, West Africa

## Abstract

Peste des petits ruminants (PPR) is a highly contagious viral disease affecting sheep and goats, causing substantial economic losses in endemic countries. In the Savannah district of Côte d’Ivoire, knowledge of the genetic diversity and molecular epidemiology of the PPR virus (PPRV) remains limited. This study investigated the genetic diversity and phylogenetic relationships of PPRV circulating in this region using whole‐genome sequencing (WGS). A cross‐sectional survey was conducted between September and December 2023. Nasal swabs collected from sheep and goats were screened for PPRV ribonucleic acid (RNA) using real‐time reverse transcription polymerase chain reaction (RT‐qPCR). Samples with low quantification cycle (Cq) values of less than 35 and successful multiplex PCR amplification profiles were selected for sequencing using the Oxford Nanopore MinION platform. Near‐complete consensus genomes were generated through reference‐based assembly and analysed alongside representative strains from all recognised PPRV lineages. Of the 355 samples analysed, 25 (7.0%) tested positive for PPRV RNA, with positive detections in all three surveyed regions (Poro, Tchologo and Bagoué). The four samples with the lowest Cq values, originating from all three administrative regions, were successfully sequenced, generating genomes that covered 82.0%–86.2% of the reference genome at a depth of ≥ 10 ×. The missing regions were mainly located at the 5′ and 3′ genomic termini, as well as in limited internal regions associated with amplicon dropout. Phylogenetic analysis revealed that all four sequences belonged to lineage IV and exhibited high nucleotide similarity (98.1%–99.9%). The Ivorian strains clustered with recent lineage IV viruses from West, North and Central Africa, whereas historical Ivorian lineages I and II formed distinct clades. These findings confirm the predominance of lineage IV in northern Côte d’Ivoire and provide baseline genomic data to support molecular epidemiological surveillance in the region.

## 1. Introduction

Peste des petits ruminants (PPR) is a highly contagious transboundary viral disease primarily affecting domestic small ruminants, particularly sheep and goats [1, 2]. The disease is caused by the PPR virus (PPRV), which is currently classified as *Morbillivirus caprinae* [3, 4], within the family *Paramyxoviridae* [5, 6]. From both clinical and antigenic perspectives, PPRV is closely related to other morbilliviruses, including rinderpest virus (RPV), measles virus, canine distemper virus, phocine distemper virus and the morbilliviruses of dolphins and porpoises, with RPV being its closest genetic relative [1, 5, 7].

The PPRV genome consists of a single‐stranded, negative‐sense ribonucleic acid (RNA) of approximately 16 kilobases (kb) in length [8–10]. It encodes six structural proteins: nucleoprotein (N), phosphoprotein (P), matrix protein (M), fusion protein (F), haemagglutinin (H) and polymerase (L). In addition, two non‐structural proteins, C and V, are expressed from alternative open reading frames within the P gene. The viral genomic organisation follows the conserved gene order 3′‐N‐P/C/V‐M‐F‐H‐L‐5′, with individual genes separated by short intergenic regions [11, 12].

Traditionally, phylogenetic analyses have been based on partial nucleotide sequences of the N, F and H genes to classify PPRV into four genetically distinct lineages (I–IV) [13–15]. Lineages I, II and III were historically largely restricted to Africa, whereas lineage IV was initially reported mainly in Asia and the Middle East [1, 16, 17]. However, over the past two decades, lineage IV has expanded extensively across Africa, progressively replacing the other lineages and becoming dominant in many endemic regions. The earliest reports of lineage IV in Africa date back to the early 2000s, with detections in countries such as Eritrea, Morocco and Sudan [18, 19]. In West Africa, lineage IV was first documented in Nigeria, in samples collected from 2010 onwards [16, 20], suggesting it was introduced to the region relatively recently. Since then, it has been increasingly reported in several other West African countries, including Côte d’Ivoire, as well as in neighbouring countries such as Burkina Faso, Ghana, Guinea and Mali [21–23]. The progressive detection of lineage IV in multiple countries indicates a gradual spread, likely facilitated by transboundary livestock movements and pastoral trade networks.

Transmission of PPRV occurs rapidly, primarily through direct contact between infected and susceptible animals via respiratory secretions and excretions, including nasal and ocular discharges, saliva, urine and faeces [24–26]. Inhalation of virus‐laden aerosols expelled during coughing or sneezing represents the primary route of infection. Although indirect transmission via contaminated fomites, such as bedding, water troughs or feeding equipment, has been reported, it is considered of secondary importance [25, 27]. In immunologically naïve populations, morbidity can approach 100%, with mortality rates typically ranging from 70% to 90% [8, 25]. Clinical manifestations include high fever, nasal and ocular discharges, necrotic oral lesions, severe diarrhoea and respiratory distress, with disease severity influenced by both host‐related and environmental factors [2, 25, 28].

Although PPR primarily affects sheep and goats, PPRV infection has been documented in a range of other domestic and wild species. Among domestic animals, infections have been reported in cattle (*Bos taurus*), camels (*Camelus dromedarius*) and domestic pigs (*Sus scrofa domesticus*) [29–32]. In wildlife, several ungulate species, including African buffalo (*Syncerus caffer*), gazelles (*Gazella* spp.), saiga antelope (*Saiga tatarica*) and cervids such as red deer (*Cervus elaphus*), fallow deer (*Dama dama*) and roe deer (*Capreolus capreolus*), have been identified as susceptible hosts, raising concerns regarding their role in viral maintenance and transmission [33–36].

First reported in Côte d’Ivoire in 1942 [37], PPR is now recognised as a major constraint to small ruminant production across Africa, Asia and the Middle East [1, 38]. The disease is currently present in more than 70 countries and is estimated to cause economic losses approaching US$2billion annually in endemic countries [39]. Beyond its direct impact on animal health and productivity, PPR severely affects the livelihoods of smallholder farmers and pastoralists, exacerbating poverty and food insecurity and disrupting regional and international trade through restrictions on livestock movements [39, 40].

In response to these challenges, the Food and Agriculture Organization (FAO) of the United Nations and the World Organisation for Animal Health (WOAH) have designated PPR as a notifiable animal disease and launched a global eradication strategy aiming to eliminate it by 2030 [39]. This strategy relies on mass vaccination, strengthened epidemiological and molecular surveillance, movement control, improved biosecurity and reinforced veterinary services and community engagement [41]. However, despite the availability of safe and effective live attenuated vaccines, their implementation remains limited due to logistical, economic and sociocultural constraints. Consequently, detailed investigations into the genetic diversity and molecular epidemiology of PPRV remain essential, particularly in West Africa, where viral circulation dynamics are still insufficiently characterised [42].

Côte d’Ivoire and its neighbouring countries, including Mali, Burkina Faso, Ghana, Guinea and Liberia, lie within a highly endemic zone for PPR, where small ruminants play a central role in pastoral and agropastoral systems. Frequent cross‐border livestock movements associated with trade and seasonal transhumance, combined with limited surveillance capacity and heterogeneous vaccination coverage, continue to hinder effective disease control. In this context, the present study aimed to perform genomic characterisation and lineage assignment of PPRV circulating in the Savannah district of northern Côte d’Ivoire using next‐generation sequencing and comparative genomic analyses. By generating and analysing viral sequence data, the work seeks to improve understanding of local and regional PPRV circulation dynamics and to support evidence‐based strategies for strengthened surveillance, control and eventual eradication.

## 2. Materials and Methods

### 2.1. Study Area and Study Design

**FIGURE 1:**
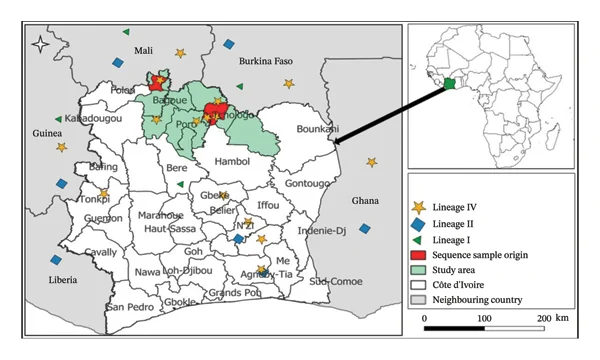
Map showing the distribution of peste des petits ruminants virus (PPRV) lineages in Côte d’Ivoire (white) and its neighbouring countries (grey). The Savannah district is highlighted in green. The red areas indicate the locations from which the PPRV‐positive samples analysed in this study were collected. Previously reported PPRV lineages in Côte d’Ivoire and neighbouring countries are indicated by coloured symbols: orange stars for lineage IV, blue squares for lineage II, and green triangles for lineage I. The map was produced using QGIS software (Version 3.38) based on administrative boundary datasets obtained from the United Nations Office for the Coordination of Humanitarian Affairs–Humanitarian Data Exchange (OCHA‐HDX).

Nasal samples were collected from sheep and goats during a cross‐sectional survey conducted between September and December 2023 in the Savannah district of northern Côte d’Ivoire (Figure 1). The district comprises three administrative regions (Poro, Tchologo and Bagoué) and shares international borders with Burkina Faso and Mali. Owing to its geographic location, the Savannah district serves as a major entry corridor for commercial livestock and transhumant animals from Sahelian countries, particularly Burkina Faso and Mali. In each of the three regions, two departments were randomly selected. Within each department, three subprefectures were selected, followed by the random selection of three villages from official lists provided by the local veterinary authorities. In each selected village, 16 unvaccinated small ruminants, as reported by the attending veterinary officer and/or the animal owner, were sampled. Four animals per farm were selected irrespective of their clinical status. Eligible animals were at least 6 months of age, with age estimated by dentition, and included both males and females.

Previous serological investigations have reported high levels of PPRV exposure in this area. In Korhogo, the administrative capital of the district, PPRV seroprevalence was estimated at 36.6% in 2020 [43], comparable to the seroprevalence reported in neighbouring countries such as Burkina Faso (36.7% and 28.9%) [44, 45] and Mali (32% and 42.6%) [46, 47]. These findings highlight the epidemiological importance of the Savannah district as a high‐risk zone for PPRV circulation.

### 2.2. Sample Collection

**FIGURE 2:**
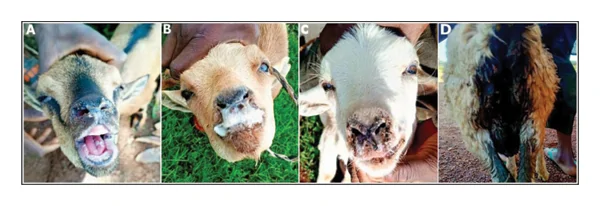
Clinical signs consistent with peste des petits ruminants (PPR) observed in small ruminants from which samples were collected in northern Côte d’Ivoire in 2023. (A) Oral lesions associated with buccal and nasal discharges in a goat; (B, C) Mucopurulent nasal discharge in goats showing signs of clinical deterioration; (D) Severe diarrhoea in a sheep. Animals shown in panels (A), (B) and (C) were sampled in the Sinématiali Department (Poro region), while the animal in panel (D) was sampled in the Boundiali Department (Bagoué region). The presence of PPR virus (PPRV) was confirmed in all corresponding samples by molecular analyses by real‐time reverse transcription PCR (RT‐qPCR) and conventional PCR.

Nasal swab samples were collected in accordance with the WOAH guidelines for PPR surveillance [48]. Samples were obtained from live sheep and goats irrespective of the presence or absence of clinical signs suggestive of PPR, including fever, diarrhoea, lacrimation, conjunctivitis and nasal or ocular discharge (Figure 2).

Sterile cotton‐tipped swabs mounted on flexible shafts were moistened with viral transport medium (VTM), inserted into the nasal cavity, and gently rotated against the nasal mucosa for approximately 1 min. Each swab was immediately placed into a sterile tube containing 2 mL of VTM, uniquely labelled and maintained at 4°C on ice during transport to the Korhogo Regional Laboratory (LRK) under the supervision of the National Laboratory for Agricultural Development Support (LANADA).

Upon arrival, samples were stored at −80°C until transfer to the Central Veterinary Laboratory of Bingerville (LCVB/LANADA) for molecular analyses. Before RNA extraction, samples were vortexed and centrifuged at 5000 revolutions per minute (rpm) for 10 min at 4°C, and the resulting supernatants were collected and stored at −80°C.

### 2.3. RNA Extraction, Complementary Deoxyribonucleic Acid (cDNA) Synthesis and Sample Selection

Total RNA was extracted from nasal swab supernatants using the RNeasy Mini Kit (QIAGEN GmbH Cat. No. 74106, Hilden, Germany) in accordance with the manufacturer’s instructions. Detection of PPRV RNA was performed using real‐time reverse transcription polymerase chain reaction (RT‐qPCR) targeting the PPRV N gene, according to the protocol described by Batten et al. [49]. The primers and probe used were PPRNF (5′‐AGAGTTCAATATGTTRTTAGCCTCCAT‐3′), PPRN (5′‐TTCCCCARTCACTCTYCTTTGT‐3′), and PPRN probe (FAM‐5′‐CACCGGAYACKGCAGCTGACTCAGAA‐3′‐TAMRA).

The quantification cycle (Cq) values from RT‐qPCR were used as a measure of viral RNA load and as the main criterion for choosing samples for whole‐genome sequencing (WGS). Lower Cq values correspond to higher viral genome copy numbers and an increased likelihood of successful amplification in later steps. Following RT‐qPCR screening, samples with low Cq values (< 35) were selected for multiplex tiling PCR amplification.

Only samples selected for downstream analysis underwent cDNA synthesis. For genome amplification, cDNA was synthesised using the SuperScript III First‐Strand Synthesis System (Invitrogen Cat. No 18091200, MA, USA), following the manufacturer’s recommendations. Briefly, 8 μL of extracted RNA was subjected to reverse transcription at 23°C for 10 min, followed by 55°C for 15 min, with subsequent enzyme inactivation at 80°C for 10 min. Residual RNA was then removed by adding 1 μL of RNase H and incubating at 37°C for 20 min.

### 2.4. Whole‐Genome PCR Amplification

Whole‐genome amplification was performed using Q5 Hot Start High‐Fidelity DNA Polymerase (New England BioLabs Cat. No. M0493L, Ipswich, MA, USA) and a multiplex primer scheme generating overlapping amplicons of approximately 800 bp, as previously described by Torsson & Lindsjö [50]. A total of 44 primer pairs were used, divided into two pools of 22 primer pairs each. Primers were prepared at a working concentration of 10 μM and were designed based on eight representative full‐genome PPRV sequences covering all known viral lineages.

The primer sets generated overlapping amplicons with an average overlap of approximately 100 bp across the PPRV genome. Primer pool 1 comprised odd‐numbered primer pairs, whereas Primer pool 2 contained even‐numbered primer pairs. Conventional PCR amplification was performed using an Eppendorf Mastercycler Nexus Gradient PCR System (Eppendorf AG, Hamburg, Germany) under the cycling conditions described by Torsson & Lindsjö [50]. Amplicons from both primer pools were combined and purified using AMPure XP magnetic beads (Beckman Coulter Life Sciences Cat. No. A63AA0, Brea, CA, USA) at a bead‐to‐sample ratio of 1.8×.

### 2.5. Amplicon Quantification and Quality Control

Purified PCR products were quantified using the Qubit dsDNA High Sensitivity Assay Kit (Thermo Fisher Scientific Cat. No. Q32854, Waltham, MA, USA) on a Qubit 1.0 fluorometer, according to the manufacturer’s instructions. Amplicon quality and size were assessed by electrophoresis on a 1.5% agarose gel. Briefly, 5 μL of each purified DNA was loaded into an individual well, with a DNA size marker in a separate well. Electrophoresis was performed at 100 V for 30 min, after which DNA fragments were visualised under ultraviolet illumination using a Gel Doc EZ Imager system (Bio‐Rad Laboratories, Hercules, CA, USA).

Sample selection for WGS was prioritised based on the quality of multiplex tiling PCR amplification. Amplification products were visualised by agarose gel electrophoresis, and samples showing strong amplicon profiles of the expected size (∼800 bp) were selected for Oxford Nanopore sequencing.

### 2.6. Nanopore Library Preparation and Sequencing

Purified multiplex PCR products were diluted to a final concentration of 0.12 pmol in a total volume of 25 μL of nuclease‐free water. End‐repair and A‐tailing were performed according to the Oxford Nanopore Technologies (ONT) protocol, followed by magnetic bead purification and elution in 15 μL of nuclease‐free water. DNA concentration was reassessed using the Qubit dsDNA High Sensitivity Assay Kit to ensure accurate quantification before native barcoding and to confirm that the recommended input amount for library preparation was achieved.

Native barcoding was performed according to ONT recommendations, and barcoded samples were pooled equimolarly to obtain a final library concentration of 0.15 pmol. Sequencing adapters were ligated, followed by bead purification and final elution in 15 μL of sequencing‐ready library.

Sequencing was performed on an Oxford Nanopore MinION MK1c device using an R9.4.1 flow cell. Sequencing reagents, including sequencing buffer (SQB), loading beads (LB), flow cell tether (FLT) and flow cell flush buffer (FLB), were thawed and kept on ice until further use. The flow cell was primed with 800 μL of an FLB‐FLT mixture, followed by a 5‐min equilibration period and the addition of a further 200 μL of priming mix. Finally, 75 μL of the prepared library was loaded dropwise into the SpotON sample port, and sequencing was initiated using MinKNOW software and run for 10 h.

### 2.7. Sequence Analysis and Consensus Genome Quality Assessment

Raw Oxford Nanopore sequencing reads were processed under WSL‐Ubuntu 22.04 using MobaXterm (Personal Edition v25.2, Build 5296) within a dedicated Conda environment configured for PPRV whole‐genome analysis. Initial sequencing quality metrics, including read length distribution, sequencing yield and quality score profiles, were assessed using NanoPlot (v1.46.1).

For each sample, all generated FASTQ files were concatenated into a single file before adapter trimming with Porechop (v0.2.4). Sequencing reads were then filtered with Filtlong (v0.2.1), retaining sequences ≥ 500 bp and the top 90% of the highest quality reads. Postfiltering quality metrics were reassessed using NanoPlot to confirm improvements in read length distribution and quality.

Filtered reads were aligned to the PPRV lineage IV reference genome (NC_006383) using Minimap2 v2.28 with the map‐ont preset. Alignment files were sorted and indexed using Samtools v1.20. Variant calling was performed using bcftools mpileup and bcftools call (v1.20), and consensus genome sequences were generated using bcftools consensus, applying a minimum allele frequency threshold of 0.6 and retaining positions supported by a sequencing depth of ≥ 10 ×.

Genome completeness was assessed as the proportion of the reference genome covered at ≥ 10 × depth, together with evaluation of mean sequencing depth and coverage uniformity across the genome. Regions of low or zero coverage, ambiguous base calls (N positions) and sequence gaps were identified by alignment of consensus sequences to the reference genome. Consensus genome quality was evaluated using a combination of quantitative and qualitative criteria, including: (i) genome coverage at ≥ 10 × depth, (ii) high mean sequencing depth across covered regions, (iii) a limited proportion of ambiguous bases, and (iv) consistency of consensus base calls supported by aligned reads. All regions failing to meet these criteria were manually inspected using Integrative Genomics Viewer (IGV, v2.16.0) to assess read depth, strand balance and base‐call support. Positions showing insufficient coverage or conflicting nucleotide signals were masked or excluded from downstream analyses. Only consensus genomes meeting these predefined quality thresholds were retained for phylogenetic and comparative genomic analyses.

### 2.8. Phylogenetic Analysis

Consensus genome sequences generated were compared against the National Centre for Biotechnology Information (NCBI) (https://www.ncbi.nlm.nih.gov/genbank/) nucleotide database using the Basic Local Alignment Search Tool for nucleotides (BLASTn) (https://blast.ncbi.nlm.nih.gov/Blast.cgi) to identify closely related PPRV reference sequences. Relevant metadata, including country of origin, host species and year of collection, were retrieved and incorporated into the dataset.

The final dataset comprised four near‐complete PPRV genome sequences generated in this study and 27 complete reference genomes, representing PPRV lineages I–IV, originating from West Africa, North Africa, Central Africa, East Africa and Asia. Multiple sequence alignment was performed using MAFFT (Multiple Alignment using Fast Fourier Transform, v7.475) (https://mafft.cbrc.jp/alignment/server/) under default parameters. The resulting alignment was visually inspected using BioEdit (Biological Sequence Alignment Editor, v7.2.5) (https://bioedit.software.informer.com/) to assess alignment quality. Poorly aligned regions and ambiguous nucleotide positions were trimmed, primarily corresponding to regions of incomplete genome coverage.

Phylogenetic reconstruction was performed using the maximum likelihood (ML) method implemented in MEGA X (Molecular Evolutionary Genetics Analysis, Version X) (https://www.megasoftware.net/) [51]. The optimal nucleotide substitution model was selected within MEGA X, and the General Time Reversible model with gamma‐distributed rate variation among sites (GTR + G) was used for tree inference. Statistical support for internal branches was evaluated using 1000 bootstrap replicates, and bootstrap values below 50% were not displayed on the final phylogenetic tree. Phylogenetic tree visualisation and annotation were performed within the MEGA X environment.

## 3. Results

### 3.1. Detection of PPRV and Sample Selection for Whole‐Genome Sequencing

A total of 355 nasal swab samples were collected from small ruminants during the cross‐sectional survey, including 192 goats (54.1%) and 163 sheep (45.9%). Samples originated from three administrative regions of the Savannah district: Poro (*n* = 132; 37.2%), Bagoué (*n* = 115; 32.4%) and Tchologo (*n* = 108; 30.4%). The sampled population was predominantly female (*n* = 237; 66.8%), with 118 males (33.2%). Animals were distributed across three age categories: < 1 year (*n* = 103; 29.0%), 1–2 years (*n* = 121; 34.1%) and > 2 years (*n* = 131; 36.9%).

Molecular screening by RT‐qPCR detected PPRV RNA in 25 of 355 samples (7.0%), comprising 13 of 192 goats (6.8%) and 12 of 163 sheep (7.4%). A higher proportion of positives were identified among females (*n* = 18/237; 7.6%) than among males (*n* = 7/118; 6.0%). Positive samples were detected in all three regions, including Poro (*n* = 10/132; 7.6%), Bagoué (*n* = 8/115; 7.0%) and Tchologo (*n* = 7/108; 6.5%) (Supporting Table S1).

Confirmatory testing using conventional RT‐PCR (RT‐cPCR), which targeted the N gene, identified 17 positive samples, including 11 goats and six sheep. Among these, 14 samples originated from female animals and three from males. The regional distribution included Poro (*n* = 7), Bagoué (*n* = 7) and Tchologo (*n* = 3) (Supporting Table S1).

**FIGURE 3:**
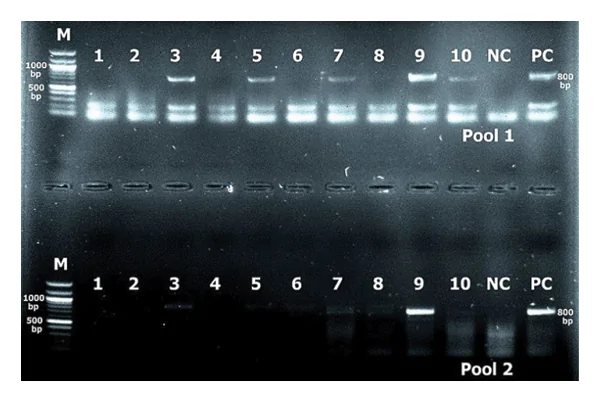
Agarose gel electrophoresis of multiplex tiling polymerase chain reaction (PCR) products generated for whole‐genome amplification of the peste des petits ruminants virus (PPRV) from field samples collected in the Savannah district, northern Côte d’Ivoire, in 2023. Two multiplex primer pools (Pool 1 and Pool 2) were used to amplify overlapping ∼800 bp fragments spanning the PPRV genome. Strong amplification of the expected ∼800 bp products was observed for Samples 3, 5, 7 and 9. Samples 3 and 5 originated from Sinématiali (Poro region), Sample 7 from Ferkessédougou (Tchologo region), and Sample 9 from Tengréla (Bagoué region). Additional low‐molecular‐weight bands (∼100–200 bp), observed mainly in Pool 1, are most likely attributable to primer‐dimer formation or non‐specific primer artefacts and were not considered suitable for sequencing. M: 100‐bp molecular weight marker; NC: negative control; PC: positive control; 1–10: analysed samples; bp: base pairs.

For WGS, RT‐cPCR‐positive samples with the lowest RT‐qPCR Cq values were prioritised. Following multiplex tiling PCR amplification, samples that produced strong and specific amplicon profiles of the expected size (∼800 bp) were selected for sequencing. Based on these criteria, 10 samples were initially retained as candidates for WGS. However, only four samples that generated high‐quality amplification products were selected for sequencing (Figure 3). These samples originated from Sinématiali (Poro, *n* = 2), Ferkessédougou (Tchologo, *n* = 1) and Tengréla (Bagoué, *n* = 1) (Table 1).

**TABLE 1:** Characteristics of the ten PPRV‐positive samples collected in the Savannah district, northern Côte d’Ivoire, in 2023 and selected as candidates for whole‐genome sequencing based on low RT‐qPCR Cq values and successful multiplex tiling PCR amplification.

Location	Species	Breed	Age	Sex	RT‐cPCR (selected)	Successfully sequenced
*Tchologo region*						
Ferkessédougou	Goat	WAD	> 2 years	Female	+	+
	Goat	WAD	< 2 years	Female	+	−

*Poro region*						
Sinématiali	Goat	WAD	< 1 year	Female	+	+
	Goat	WAD	> 2 years	Female	+	+
	Goat	WAD	< 2 years	Female	+	−

Korhogo	Sheep	WAD	< 2 years	Male	+	−

*Bagoué region*						
Tengréla	Goat	WAD	> 2 years	Female	+	+
	Sheep	WAD	> 2 years	Female	+	−

Boundiali	Goat	WAD	< 2 years	Male	+	−
	Goat	WAD	< 1 year	Female	+	−

*Note:* RT‐qPCR, real‐time reverse transcription polymerase chain reaction.

Abbreviations: Cq, quantification cycle; PCR, polymerase chain reaction; PPRV, peste des petits ruminants virus; RT‐cPCR, reverse transcription conventional polymerase chain reaction; WAD, West African Dwarf.

All sequenced samples were obtained from West African Dwarf (WAD) goats, including one animal aged < 1 year and three aged > 2 years. Some animals selected exhibited clinical signs consistent with PPR, including nasal discharge and diarrhoea (Figure 2).

### 3.2. Nanopore Read Production and Initial Quality Assessment

Oxford Nanopore sequencing of the four selected samples yielded sufficient data for downstream analyses. Basic sequencing run statistics, including read numbers, sequencing yield, read length distribution and quality metrics, are summarised in Table 2.

**TABLE 2:** Summary of Oxford Nanopore sequencing output, genome assembly metrics, sequencing depth, genome coverage and amplicon‐associated gaps for near‐complete peste des petits ruminants virus (PPRV) sequences generated from samples collected in the Savannah district, northern Côte d’Ivoire in 2023.

Sample ID	Raw reads	Total bases	N50 length (bp)	PPRV‐mapped reads	Mean read quality (Q)	Mean depth	Consensus length (bp)	Average coverage (%)	GC content (%)	Amplicon‐associated gaps
B1	519,496	183,605,665	703	49,893	9.3	2011×	13,278	83.26	48.58	1, 9, 11, 24, 25
B2	373,681	140,994,262	702	43,963	9.3	1765×	13,156	82.50	48.59	1, 9, 11, 24, 25
T1	796,479	285,432,493	715	42,730	9.3	1667×	13,744	86.20	48.15	1, 9, 11, 24, 25
M1	660,226	251,999,334	712	32,948	9.4	1314×	13,079	82.01	48.64	1, 9, 11, 24, 25

*Note:* B1: PPRV/Côte_d’Ivoire/2023/B1; B2: PPRV/Côte_d’Ivoire/2023/B2; T1: PPRV/Côte_d’Ivoire/2023/T1; M1: PPRV/Côte_d’Ivoire/2023/M1.

Abbreviations: bp, base pairs; PPRV, peste des petits ruminants virus.

### 3.3. Consensus Genome Generation and Gaps Analysis

Near‐complete consensus genome sequences were successfully generated for all four samples following read filtering, reference‐based mapping and variant calling. Final consensus lengths ranged from 13,079 bp (PPRV/Côte_d’Ivoire/2023/M1) to 13,744 bp (PPRV/Côte_d’Ivoire/2023/T1). Genome coverage at ≥ 10 × depth was 86.20% for PPRV/Côte_d’Ivoire/2023/T1, 83.26% for PPRV/Côte_d’Ivoire/2023/B1, 82.50% for PPRV/Côte_d’Ivoire/2023/B2 and 82.01% for PPRV/Côte_d’Ivoire/2023/M1, relative to the PPRV lineage reference genome. Coverage gaps were primarily located at the 5′ and 3′ genomic termini and in limited internal regions corresponding to amplicon dropout.

**FIGURE 4:**
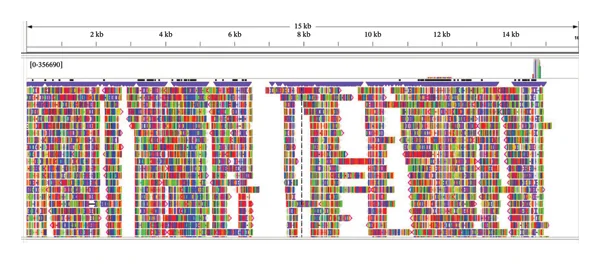
Integrative Genomics Viewer (IGV) visualisation of Oxford Nanopore sequencing reads from PPRV/Côte_d’Ivoire/2023/B1 aligned to the reference PPRV genome (GenBank Accession no. NC_006383). The alignment illustrates sequencing read depth and genome coverage across the viral genome. Coloured bars represent individual aligned sequencing reads. Coverage gaps correspond to genomic regions with insufficient or no sequencing reads.

Visual inspection of genome coverage profiles using IGV revealed multiple regions of low or absent read coverage corresponding to gaps in the assembled genomes (Figure 4). Each consensus genome contained five coverage gaps (Table 2).

### 3.4. Phylogenetic Analysis

**FIGURE 5:**
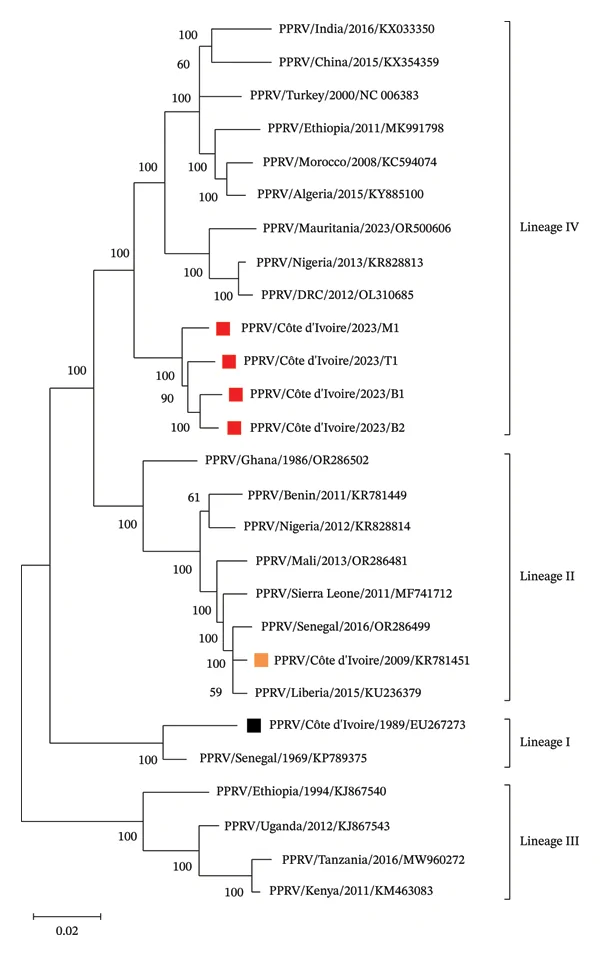
Maximum‐likelihood (ML) phylogenetic tree inferred from near‐complete genome nucleotide sequences of peste des petits ruminants virus (PPRV). The tree includes the four PPRV sequences generated in this study from the Savannah district of northern Côte d’Ivoire in 2023 (highlighted by a red square) together with selected sequences retrieved from GenBank. Phylogenetic reconstruction was performed in MEGA X using the ML method under the general time‐reversible model with gamma‐distributed rates among sites (GTR + G) (Kimura, 1980). Bootstrap values were estimated from 1000 replicates, and values below 50% were omitted. The previously identified lineage II sequence from Côte d’Ivoire is shown as a solid orange square and the lineage I sequence as a solid black square. Sequences from countries neighbouring Côte d’Ivoire were included for phylogenetic comparison. Representative sequences from neighbouring West African countries were included to facilitate regional phylogenetic comparison.

ML phylogenetic analysis incorporating the four near‐complete Ivorian genomes and representative reference sequences from all four PPRV lineages demonstrated that all Ivorian sequences described in this study clustered within lineage IV (Figure 5; Supporting Table S2). The four sequences formed a well‐supported monophyletic clade with short internal branch lengths, indicating limited genetic divergence.

Pairwise nucleotide identity among the Ivorian sequences ranged from 98.1% to 99.9%. The sequences PPRV/Côte_d’Ivoire/2023/B1, B2 and T1 were highly similar (≥ 99.9%), whereas PPRV/Côte_d’Ivoire/2023/M1 exhibited slightly lower identity values (≥ 98.1%) (Table 2). BLASTn similarity searches indicated that the most closely related sequences corresponded to lineage IV strains previously reported in West, North and Central Africa, including isolates from Nigeria (2013), Mauritania (2023), Morocco (2008), Algeria (2015) and the Democratic Republic of the Congo (2012).

## 4. Discussion

This study provides molecular evidence of the active and widespread circulation of PPRV in the Tchologo, Poro and Bagoué regions of Côte d’Ivoire’s Savannah district in the north of the country. This area plays a central role in national and regional livestock systems, with high densities of small ruminants and intensive animal movements. Northern Côte d’Ivoire is a major entry point for livestock from neighbouring Sahelian countries, especially Burkina Faso and Mali [52, 53]. Seasonal transhumance and commercial trade contribute to the large‐scale, annual movement of animals across borders, which is facilitated by regional mobility agreements within the framework of the Economic Community of West African States (ECOWAS) [54, 55]. This type of livestock movement has been identified as a key factor in the spread of transboundary animal diseases in West Africa [56, 57].

The detection of PPRV RNA by real‐time RT‐PCR in samples collected across all three regions suggests sustained viral circulation in this hyperendemic setting. The broad geographic distribution of positive cases indicates that transmission is ongoing and widespread rather than being restricted to isolated or spatially limited outbreaks. In endemic areas such as northern Côte d’Ivoire, where partial herd immunity, subclinical infections and heterogeneous viral loads may fluctuate, sensitive molecular diagnostics are essential for accurately characterising transmission dynamics and strengthening surveillance systems.

All sequenced samples originated from West African Dwarf goats, a breed widely recognised as highly susceptible to PPRV infection [8, 25, 58]. The detection of infection in both young and adult animals, along with the widespread geographic distribution of positive cases, suggests continuous viral transmission across age groups rather than isolated or short‐lived outbreaks. When considered alongside the wide spatial dispersion of positive cases, these findings are consistent with frequent animal movements within and across administrative boundaries, which likely facilitate the virus’s dissemination within the Savannah district.

Near‐complete genome sequencing revealed that all PPRV sequences collected in Côte d’Ivoire in 2023 were exclusively from lineage IV. The findings of this study specifically reflect PPRV circulation in the Savannah district of northern Côte d’Ivoire and should not be interpreted as fully representative of the viral diversity circulating throughout the country. Given the limited geographical coverage and number of genomes analysed, additional investigations from other regions of Côte d’Ivoire are required to provide a more comprehensive picture of national PPRV diversity and population structure. This finding lends weight to the theory that this lineage is currently dominant in the region. This corroborates previous reports documenting lineage IV’s gradual replacement of historical lineages I and II in West Africa over the past two decades, particularly in Côte d’Ivoire, Niger, Burkina Faso, Mali, Guinea, Ghana, Nigeria and Senegal [16, 17, 20, 22, 45, 59, 60]. The high nucleotide identity observed among the Ivorian sequences (98.1%–99.9%) suggests the homogeneous circulation of lineage IV variants in northern Côte d’Ivoire. However, this apparent genetic similarity should be interpreted with caution, as some of the observed similarities may be due to technical constraints associated with Nanopore‐only sequencing. These include variable sequencing depth, incomplete genome coverage and coverage gaps, all of which may reduce the detection of low‐frequency variants and fine‐scale genetic differences among closely related strains. Furthermore, the observed high nucleotide identity values should be interpreted carefully because the most variable genomic regions, particularly the 5′ and 3′ termini, were not fully represented in the analysed genomes. Consequently, the genomic similarity among sequenced isolates may be slightly overestimated relative to comparisons based on complete genome sequences.

The exclusive detection of lineage IV is nevertheless consistent with reports from neighbouring West African countries, including Burkina Faso, Ghana, Mali, Niger, Nigeria, Guinea and Senegal, where lineage IV has progressively become the predominant circulating lineage. The clustering of the Ivorian sequences with contemporary lineage IV strains from West, North and Central Africa further supports the existence of interconnected regional transmission networks shaped by livestock trade, seasonal transhumance and cross‐border animal movements.

Despite the geographical separation of sampling locations, the limited genetic divergence among the sequences is consistent with the virus spreading relatively quickly through epidemiologically connected transmission networks, rather than through multiple, clearly independent introductions. However, the available data do not allow definitive conclusions to be drawn about a single introduction pathway or source. More extensive spatial and temporal sampling, combined with fully complete genome sequences, would be required to robustly resolve introduction events and transmission routes.

Although these findings are restricted to northern Côte d’Ivoire, the phylogenetic clustering of the generated sequences with contemporary lineage IV strains from several West African countries is consistent with the broader regional circulation pattern reported over the last decade. This supports the hypothesis that northern Côte d’Ivoire forms part of an interconnected West African epidemiological network shaped by transboundary livestock movements. A comparative phylogenetic analysis revealed that the Ivorian sequences shared 93.8%–95.1% nucleotide identity with recent lineage IV strains from Mauritania (OR500606), Nigeria (KR828813), Morocco (KC594074), Algeria (KY885100) and the Democratic Republic of the Congo (OL310685). All sequences clustered within a well‐supported subclade (bootstrap support = 100%), consistent with the circulation of a genetically related lineage IV viral pool across West Africa. This phylogenetic proximity suggests the interconnected circulation of lineage IV across West, North and Central Africa, which is likely shaped by pastoral systems and commercial livestock networks, as was previously proposed by Baazizi et al. [61]. By contrast, lineage III strains from East Africa, including those from Tanzania (MW960272), Uganda (KJ867543), Kenya (KM463083) and Ethiopia (KJ867540), appear genetically more distant. This reflects the long‐standing geographical and evolutionary separation between the PPRV foci in West and East Africa. This differentiation underscores the importance of whole‐genome–based analyses for accurately reconstructing PPRV dispersal routes beyond approaches that rely solely on partial gene sequences.

Unlike phylogenetic analyses based solely on partial gene fragments, the near‐complete genome sequences generated in this study enabled inference of genome‐wide nucleotide variation. The analysed genomes covered a large proportion of the PPRV coding region, enabling higher resolution discrimination between closely related lineage IV strains. This genome‐scale approach provided strong phylogenetic support for clustering of the Ivorian sequences, clarifying their close relationship with contemporary lineage IV viruses circulating across multiple African regions. To the best of our knowledge, these data represent the first near‐complete genome sequences of contemporary PPRV strains from Côte d’Ivoire, providing a crucial update to the historical genomic data available for the country.

Against the backdrop of the global PPR eradication strategy, which aims to eliminate the disease by 2030 [62], these findings underscore the importance of strengthening molecular surveillance in endemic and hyperendemic regions. High‐resolution genomic data can improve our understanding of viral circulation patterns and transboundary transmission dynamics. The successful application of portable Oxford Nanopore sequencing in this study demonstrates its potential to enhance in‐country sequencing capacity in resource‐limited settings. Future investigations would benefit from an increased sample size, improved genome completeness, and the integration of additional short‐read sequencing technologies in order to mitigate platform‐specific biases and enable more robust evolutionary and epidemiological inferences.

### 4.1. Study Limitations

Although near‐complete genomes were obtained, some regions remained uncovered, primarily at the genomic ends and in a few internal regions associated with amplicon dropout. Additional sequencing approaches, such as targeted PCR amplification followed by Sanger sequencing, could potentially close these gaps. However, due to limited RNA quantity and resource constraints, gap‐filling sequencing was not performed in the present study. Consequently, the generated genomes were near‐complete rather than fully complete. Coverage gaps resulting from uneven sequencing depth and variable amplification efficiency may have reduced sensitivity in detecting low‐frequency variants and fine‐scale genetic differences among closely related strains. In addition, the incomplete genome coverage precluded protein‐level analyses, preventing the assessment of amino acid variation, potential functional mutations or antigenic differences.

This study has some limitations that should be considered when interpreting the results. Firstly, only four PPRV‐positive samples generated near‐complete genome sequencing suitable for downstream analysis. No formal power analysis was conducted for phylogenetic inference because the study was designed as an exploratory genomic investigation. The number of genomes available for analysis was constrained by the RT‐qPCR positivity rate observed during field sampling and by the successful recovery of near‐complete genomes from samples with adequate viral RNA loads. Consequently, the small number of sequenced genomes (*n* = 4) limits the statistical power of phylogenetic and phylogeographic investigations, and restricts the ability to infer introduction events, transmission pathways and the overall genetic diversity of PPRV circulation in northern Côte d’Ivoire.

Furthermore, the high nucleotide identity values observed among the Ivorian sequences (98.1%–99.9%) should be interpreted with caution, since the most variable genomic regions, particularly the 5′ and 3′ termini, were not fully represented in the analysed genomes. The incomplete coverage of these terminal regions, where genetic variability is often greatest, may have resulted in a slight overestimation of genetic similarity among sequenced isolates. Therefore, the reported nucleotide identity values are valid only for the genomic regions successfully recovered and may not fully reflect whole‐genome diversity.

Finally, using only Oxford Nanopore sequencing without short‐read validation using a complementary short‐read technology may have introduced platform‐specific biases that affect base‐level accuracy, including base‐calling accuracy and consensus sequence precision. Despite the application of consensus generation and quality filtering, minor sequencing errors cannot be entirely ruled out. Future studies that incorporate larger sample sizes, improved genome completeness, targeted gap‐filling approaches, and integrated long‐ and short‐read sequencing strategies would enhance the resolution of viral diversity and enable more robust evolutionary and epidemiological interpretations.

## 5. Conclusion

This study provides molecular evidence of PPRV circulation in the Savannah district of northern Côte d’Ivoire and presents the first near‐complete PPRV genome sequences from contemporary field isolates in the country. Phylogenetic analyses consistently placed all sequenced viruses within lineage IV, which supports the current predominance of this lineage in northern Côte d’Ivoire. These findings are also consistent with reports of lineage IV circulation across several other West African countries.

Comparative genome‐wide analyses demonstrated that the Ivorian viruses clustered with contemporary lineage IV strains circulating in West, North and Central Africa, highlighting the regional connectivity of PPRV populations and the transboundary nature of virus circulation. These findings contribute to the growing body of evidence documenting the widespread establishment of lineage IV across the African continent.

Importantly, this study establishes a genomic baseline for future molecular epidemiological and evolutionary research in Côte d’Ivoire. It also demonstrates the feasibility of generating near‐complete PPRV genomes using portable Oxford Nanopore sequencing technology in settings with limited resources, thereby supporting the development of in‐country genomic surveillance capacity. To better characterise PPRV diversity, track viral evolution, and strengthen evidence‐based control and eradication efforts in Côte d’Ivoire and the wider West African region, expanded genomic surveillance incorporating broader spatial and temporal sampling, improved genome completeness, and complementary sequencing approaches will be essential.

## Author Contributions

Conceptualisation: Konan Ange‐Sylvestre Goli; data curation: Konan Ange‐Sylvestre Goli, Jean Nepomuscene Hakizimana, Charlie Franck Amoia and Mariam Makange; formal analysis: Konan Ange‐Sylvestre Goli and Jean Nepomuscene Hakizimana; funding acquisition: Gerald Misinzo; investigation: Konan Ange‐Sylvestre Goli; methodology: Konan Ange‐Sylvestre Goli, Jean Nepomuscene Hakizimana, Augustino Alfred Chengula, Kiffopan Benjamin M’Bari, Melvyn Quan, Peter N. Thompson and Gerald Misinzo; project administration: Gerald Misinzo; software: Konan Ange‐Sylvestre Goli; supervision: Augustino Alfred Chengula, Melvyn Quan, Peter N. Thompson and Gerald Misinzo; validation: Augustino Alfred Chengula, Melvyn Quan, Peter N. Thompson and Gerald Misinzo; visualisation: Konan Ange‐Sylvestre Goli, Jean Nepomuscene Hakizimana, Augustino Alfred Chengula, Kiffopan Benjamin M’Bari, Charlie Franck Amoia, Melvyn Quan, Mariam Makange, Peter N. Thompson and Gerald Misinzo; writing–original draft preparation: Konan Ange‐Sylvestre Goli; writing–review and editing: Konan Ange‐Sylvestre Goli, Jean Nepomuscene Hakizimana, Augustino Alfred Chengula, Kiffopan Benjamin M’Bari, Charlie Franck Amoia, Melvyn Quan, Mariam Makange, Peter N. Thompson and Gerald Misinzo.

## Funding

This study was supported by the Partnership for Skills in Applied Sciences, Engineering and Technology (PASET) through the Regional Scholarship and Innovation Fund (RSIF) doctoral scholarship awarded to Konan Ange‐Sylvestre Goli. The scholarship was administered by the SACIDS Africa Centre of Excellence for Infectious Diseases at the SACIDS Foundation for One Health, Sokoine University of Agriculture (SUA), Morogoro, Tanzania (Project Grant No. P165581).

## Disclosure

All authors have read and approved the final version of the manuscript. The corresponding author, Konan Ange‐Sylvestre Goli, confirms that this manuscript provides an honest, accurate and transparent account of the study and that no important aspects have been omitted. Any discrepancies from the original study plan have also been adequately explained. The funding bodies had no role in the design of the study; the collection, analysis or interpretation of data; the writing of the manuscript; or the decision to submit the article for publication.

## Ethics Statement

The samples used in this study were collected as part of routine epidemiological surveillance activities coordinated by the National Laboratory for Support to Agricultural Development (LANADA) in Côte d’Ivoire. Prior authorisation (N:/1090/MIRAH/DSV/SDSA) was obtained from the Regional and Departmental Directorates of the Ministry of Animal and Aquatic Resources (MIRAH) and the Department of Veterinary Services (DSV).

According to national regulations, formal institutional ethical approval is not required for livestock disease surveillance conducted by authorised veterinary services. Nevertheless, this study was conducted with the approval of the relevant authorities. Sampling was performed by licensed veterinarians during routine prevention and diagnostic campaigns. Informed consent was obtained from livestock owners before sample collection, and the study objectives were clearly explained to each farmer, who voluntarily agreed to participate in the study. No procedures were conducted beyond those required for standard surveillance and diagnostic purposes.

## Conflicts of Interest

The authors declare no conflicts of interest.

## Supporting Information

Additional supporting information can be found online in the Supporting Information section.

## Supporting information


**Supporting Information** The supporting information associated with this article includes additional tables that support the study’s results. Table S1: Characteristics of nasal swab samples testing positive for peste des petits ruminants virus (PPRV) by RT‐qPCR and RT‐cPCR in the Savannah district (northern Côte d’Ivoire), and selection criteria for sequencing. Table S2: Reference PPRV sequences retrieved from GenBank and nucleotide identity comparison with PPRV sequences generated in this study. STROBE‐Checklist_21 July 2026.

## Data Availability

The data that support the findings of this study are available from the corresponding author upon reasonable request.
