# Supplementary material for: Near‐Whole‐Genome Sequencing of Peste Des Petits Ruminants Virus Lineage IV From the Savannah District, Northern Côte d’Ivoire in 2023
Source: Vet Med Int. 2026 Sep 2;2026:6654668. doi: 10.1155/vmi/6654668 (PMC13535997; doi:10.1155/vmi/6654668)
Supplement: Supplementary file 1 [file VMI-2026-6654668-s001.zip › Goli et al._PPRV CIV_WGS_Supplementary Tables_Vet Med Inter_21 Jul 2026.docx]

# Supplementary tables

| **Location** | **Species** | **Breed** | **Age** | **Sex** | **RT-qPCR** | **RT-cPCR** | **Selected for sequencing** |
| --- | --- | --- | --- | --- | --- | --- | --- |
| **Tchologo region** |  |  |  |  |  |  |  |
| Ferkessédougou | Sheep | Sahelian | >2 years | Male | + | - | - |
|  | Goat | WAD | >2 years | Female | + | + | Yes |
|  | Goat | WAD | <2 years | Female | + | + | Yes |
|  | Sheep | Sahelian | >2 years | Male | + | - | - |
|  | Sheep | WAD | >2 years | Male | + | - | - |
| Ouangolodougou | Sheep | WAD | >2 years | Female | + | - | - |
|  | Sheep | WAD | >2 years | Female | + | + | No |
| **Poro region** |  |  |  |  |  |  |  |
| Korhogo | Sheep | WAD | <2 years | Male | + | + | Yes |
|  | Sheep | WAD | <2 years | Female | + | - | - |
|  | Sheep | WAD | >2 years | Male | + | + | No |
|  | Goat | WAD | <1 year | Male | + | - | - |
| Sinméatiali | Goat | WAD | <2 years | Female | + | - | - |
|  | Goat | WAD | <1 year | Female | + | + | Yes |
|  | Goat | WAD | <1 year | Female | + | + | No |
|  | Goat | WAD | <1 year | Female | + | + | No |
|  | Goat | WAD | >2 years | Female | + | + | Yes |
|  | Goat | WAD | <2 years | Female | + | + | Yes |
| **Bagoué region** |  |  |  |  |  |  |  |
| Boundiali | Goat | WAD | < 2 years | Male | + | + | Yes |
|  | Sheep | WAD | > 2 years | Female | + | - | - |
|  | Sheep | WAD | < 1 year | Female | + | + | Yes |
|  | Sheep | WAD | > 2 years | Female | + | + | No |
| Tengréla | Goat | WAD | > 2 years | Female | + | + | Yes |
|  | Goat | WAD | > 2 years | Female | + | + | No |
|  | Goat | WAD | >2 years | Female | + | + | Yes |
|  | Sheep | WAD | >2 years | Female | + | + | No |

**Table S1**: Characteristics of nasal swab samples testing positive for peste des petits ruminants virus (PPRV) by RT-qPCR and RT-cPCR in the Savannah district (northern Côte d’Ivoire), and selection criteria for sequencing

**WAD**: West African Dwarf; **RT-qPCR**: real-time reverse transcription polymerase chain reaction; **RT-cPCR**: conventional reverse transcription polymerase chain reaction. **Sample selection for sequencing:** Samples were selected for sequencing based on the presence of clear, specific and well-defined amplicon bands following RT-cPCR and agarose gel electrophoresis, indicating sufficient viral ribonucleic acid (RNA) quantity and integrity.

**Table S2:** Reference PPRV sequences retrieved from GenBank and nucleotide identity comparison with PPRV sequences generated in this study

| **Sequence ID** | **Accession number** | **Country of Origin** | **Year of Collection** | **Lineage** | **Percentage identity** | | | | **Host species** | **Reference** |
| --- | --- | --- | --- | --- | --- | --- | --- | --- | --- | --- |
|  |  |  |  |  | **B1** | **B2** | **T1** | **M1** |  |  |
| PPRV/Côte_d’Ivoire/2023/B1 | - | Côte d’Ivoire | 2023 | IV | 100 | 98.75 | 98.18 | 98.06 | Goat | This study |
| PPRV/Côte_d’Ivoire/2023/B2 | - | Côte d’Ivoire | 2023 | IV | 99.91 | 100.0 | 98.35 | 98.42 | Goat | This study |
| PPRV/Côte_d’Ivoire/2023/T1 | - | Côte d’Ivoire | 2023 | IV | 99.89 | 99.90 | 100.0 | 98.21 | Goat | This study |
| PPRV/Côte_d’Ivoire/2023/M1 | - | Côte d’Ivoire | 2023 | IV | 99.82 | 99.90 | 99.89 | 100.0 | Goat | This study |
| Turkey 2000 | NC_006383 | Turkey | 2000 | IV | 94.76 | 95.07 | 95.11 | 95.22 | Sheep | [1] |
| IND/Delhi/2016/05 | KX033350 | India | 2016 | IV | 93.66 | 93.78 | 93.83 | 94.00 | Goat | Unpublished |
| NGYO2013-2162 | KR828813 | Nigeria | 2013 | IV | 94.93 | 94.52 | 95.11 | 95.13 | Goat | [2] |
| PPRV-FY | KX354359 | China | 2015 | IV | 93.57 | 93.68 | 93.78 | 93.93 | Goat | Unpublished |
| Morocco 2008 | KC594074 | Morocco | 2008 | IV | 94.04 | 94.15 | 94.30 | 94.48 | Goat | [3] |
| PPRV/Mauritania/3_01/2023 | OR500606 | Mauritania | 2023 | IV | 94.72 | 94.32 | 94.85 | 94.88 | Goat | Unpublished |
| SRMV/Ethiopia/2011 | MK991798 | Ethiopia | 2011 | IV | 93.76 | 93.88 | 93.97 | 94.14 | Goat | Unpublished |
| S15 | KY885100 | Algeria | 2015 | IV | 94.17 | 94.27 | 94.41 | 94.56 | Goat | [4] |
| PPRV/DRC/Tshela/27/2012 | OL310685 | DRC | 2012 | IV | 94.66 | 94.27 | 94.81 | 94.85 | Goat | Unpublished |
| Benin/10/2011 | KR781449 | Benin | 2011 | II | 92.44 | 92.86 | 92.63 | 92.78 | Goat | [5] |
| PPRV/Ghana/Accra/1978 | OR286502 | Ghana | 1986 | II | 94.00 | 94.44 | 94.09 | 94.22 | Goat | [6] |
| CIV/01P/2009 | KR781451 | Côte d’Ivoire | 2009 | II | 92.47 | 92.90 | 92.67 | 92.76 | Goat | [7] |
| Lib/2015 | KU236379 | Liberia | 2015 | II | 92.39 | 92.83 | 92.60 | 92.71 | Goat | Unpublished |
| PPRV/Senegal/Kedougou/31/2016 | OR286499 | Senegal | 2016 | II | 92.26 | 92.68 | 92.46 | 92.55 | Goat | [6] |
| PPRV/Mali/Kolondieba/6/2013 | OR286481 | Mali | 2013 | II | 92.82 | 92.83 | 92.58 | 92.71 | Goat | [6] |
| NGKW2012-MSLN | KR828814 | Nigeria | 2012 | II | 92.62 | 93.02 | 92.79 | 93.05 | Goat | Unpublished |
| PPRV/Sierra Leone/048/2011 | MF741712 | Sierra Leone | 2011 | II | 92.38 | 92.78 | 92.50 | 92.63 | Goat | Unpublished |
| Tanzania/2016/Ngorongoro | MW960272 | Tanzania | 2016 | III | 87.60 | 87.85 | 87.79 | 87.82 | Goat | [8] |
| Uganda 2012 | KJ867543 | Uganda | 2012 | III | 89.08 | 89.31 | 89.27 | 89.30 | Goat | [9] |
| KN5/2011 | KM463083 | Kenya | 2011 | III | 87.83 | 88.08 | 87.98 | 88.03 | Goat | [10] |
| Ethiopia 1994 | KJ867540 | Ethiopia | 1994 | III | 89.04 | 89.34 | 89.25 | 89.35 | Goat | [9] |
| ICV | EU267273 | Côte d’Ivoire | 1989 | I | 89.83 | 90.01 | 89.93 | 90.15 | Goat | [11] |
| E32/1969 | KP789375 | Senegal | 1969 | I | 91.19 | 91.37 | 91.27 | 91.51 | Goat | [12] |

**DRC**: Democratic Republic of the Congo; **B1**: PPRV/Côte d’Ivoire/2023/B1; **B2**: PPRV/Côte d’Ivoire/2023/B2; **T1**: PPRV/Côte d’Ivoire/2023/T1; **M1**: PPRV/Côte d’Ivoire/2023/M1.

**References**

1.	Bailey D, Banyard A, Dash P, Ozkul A, Barrett T. Full genome sequence of peste des petits ruminants virus, a member of the Morbillivirus genus. *Virus Res*. 2005;110(1-2):119-124. doi:10.1016/j.virusres.2005.01.013

2.	Woma TY, Adombi CM, Yu D, et al. Co-circulation of Peste-des-Petits-Ruminants Virus Asian lineage IV with Lineage II in Nigeria. *Transbound Emerg Dis*. 2016;63(3):235-242. doi:10.1111/tbed.12387

3.	Muniraju M, El Harrak M, Bao J, et al. Complete Genome Sequence of a Peste des Petits Ruminants Virus Recovered from an Alpine Goat during an Outbreak in Morocco in 2008. *Genome Announc*. 2013;1(3):e00096-13. doi:10.1128/genomeA.00096-13

4.	Baazizi R, Mahapatra M, Clarke BD, Ait-Oudhia K, Khelef D, Parida S. Peste des petits ruminants (PPR): A neglected tropical disease in Maghreb region of North Africa and its threat to Europe. Attoui H, ed. *PLOS ONE*. 2017;12(4):e0175461. doi:10.1371/journal.pone.0175461

5.	Adombi CM, Waqas A, Dundon WG, et al. Peste Des Petits Ruminants in Benin: Persistence of a Single Virus Genotype in the Country for Over 42 Years. *Transbound Emerg Dis*. 2017;64(4):1037-1044. doi:10.1111/tbed.12471

6.	Courcelle M, Salami H, Tounkara K, et al. Comparative evolutionary analyses of peste des petits ruminants virus genetic lineages. *Virus Evol*. 2024;10(1):veae012. doi:10.1093/ve/veae012

7.	Dundon WG, Adombi CM, Kanu S, Loitsch A, Cattoli G, Diallo A. Complete Genome Sequence of a Lineage II Peste des Petits Ruminants Virus from Sierra Leone. *Genome Announc*. 2018;6(1):e01417-17. doi:10.1128/genomeA.01417-17

8.	Kinimi E, Mahapatra M, Kgotlele T, et al. Complete Genome Sequencing of Field Isolates of Peste des Petits Ruminants Virus from Tanzania Revealed a High Nucleotide Identity with Lineage III PPR Viruses. *Animals*. 2021;11(10):10. doi:10.3390/ani11102976

9.	Muniraju M, Munir M, Banyard AC, et al. Complete Genome Sequences of Lineage III Peste des Petits Ruminants Viruses from the Middle East and East Africa. *Genome Announc*. 2014;2(5):e01023-14. doi:10.1128/genomeA.01023-14

10.	Dundon WG, Kihu SM, Settypalli TBK, et al. First Complete Genome Sequence of a Lineage III Peste des Petits Ruminants Virus. *Genome Announc*. 2014;2(5):e01054-14. doi:10.1128/genomeA.01054-14

11.	Chard LS, Bailey DS, Dash P, Banyard AC, Barrett T. Full genome sequences of two virulent strains of peste-des-petits ruminants virus, the Côte d’Ivoire 1989 and Nigeria 1976 strains. *Virus Res*. 2008;136(1-2):192-197. doi:10.1016/j.virusres.2008.04.018

12.	Dundon WG, Yu D, Lô MM, Loitsch A, Diop M, Diallo A. Complete Genome Sequence of a Lineage I Peste des Petits Ruminants Virus Isolated in 1969 in West Africa. *Genome Announc*. 2015;3(3):e00381-15. doi:10.1128/genomeA.00381-15
