# Supplementary material for: Near‐Whole‐Genome Sequencing of Peste Des Petits Ruminants Virus Lineage IV From the Savannah District, Northern Côte d’Ivoire in 2023
Source: Vet Med Int. 2026 Sep 2;2026:6654668. doi: 10.1155/vmi/6654668 (PMC13535997; doi:10.1155/vmi/6654668)
Supplement: Supplementary file 1 [file VMI-2026-6654668-s001.zip › Goli et al._PPRV CIV_WGS_STROBE-Checklist_21 July 2026.doc]

STROBE Statement—checklist of items that should be included in reports of observational studies

|  | | Item No | Recommendation |
| --- | --- | --- | --- |
| **Title and abstract** | | 1 | (*a*) Indicate the study’s design with a commonly used term in the title or the abstract:  The study design is indicated in the abstract as a cross-sectional study (Line 44). |
|  |  |  | (*b*) Provide in the abstract an informative and balanced summary of what was done and what was found:  The abstract provides the background, objectives, study design, sample collection, molecular methods, principal findings and conclusions (Lines 38-61). |
| Introduction | | | |
| Background/rationale | | 2 | Explain the scientific background and rationale for the investigation being reported:  The Introduction section explains the epidemiological importance of peste des petits ruminants (PPR), the limited availability of whole-genome data from Côte d’Ivoire, and the need for genomic surveillance to support PPR global control and eradication (Lines 64-132). |
| Objectives | | 3 | State specific objectives, including any prespecified hypotheses:  The study objectives are clearly stated: to perform genomic characterisation and lineage assignment of PPR virus (PPRV) circulating in the Savannah district of northern Côte d’Ivoire using next-generation sequencing and comparative genomic analyses (Lines 132-138). |
| Methods | | | |
| Study design | | 4 | Present key elements of study design early in the paper:  The cross-sectional survey design is described at the beginning of the Materials and Methods section (Lines 146-153). |
| Setting | | 5 | Describe the setting, locations, and relevant dates, including periods of recruitment, exposure, follow-up, and data collection:  The study setting is described, including the Savannah district (comprising the Poro, Tchologo and Bagoué regions), the sampling period (September to December 2023) and the sampling locations. The laboratory analyses performed are also described (Lines 141-144). |
| Participants | | 6 | (*a*) *Cohort study*-Give the eligibility criteria, and the sources and methods of selection of participants. Describe methods of follow-up:  Not applicable.  *Case-control study*-Give the eligibility criteria, and the sources and methods of case ascertainment and control selection. Give the rationale for the choice of cases and controls:  Not applicable.  *Cross-sectional study*-Give the eligibility criteria, and the sources and methods of selection of participants:  The eligibility criteria and sampling strategy are described. Nasal swab samples were collected from sheep and goats during a cross-sectional survey. Animal selection criteria, sampling location, and sample collection procedures are described in the Materials and Methods section (Lines 141-153 & 161-164). |
|  |  |  | (*b*) *Cohort study*-For matched studies, give matching criteria and number of exposed and unexposed:  Not applicable.  *Case-control study*-For matched studies, give matching criteria and the number of controls per case:  Not applicable. |
| Variables | | 7 | Clearly define all outcomes, exposures, predictors, potential confounders, and effect modifiers. Give diagnostic criteria, if applicable:  The primary outcomes (PPRV detection and near-whole-genome sequencing), laboratory variables (real-time reverse transcription polymerase chain reaction (RT-qPCR) quantification cycle (Cq) values, conventional PCR (RT-cPCR) results, sequencing metrics), animal characteristics (species, breed, age, sex), and genomic variables were clearly defined. |
| Data sources/ measurement | | 8* | For each variable of interest, give sources of data and details of methods of assessment (measurement). Describe comparability of assessment methods if there is more than one group:  Data source and laboratory procedures are fully described, including sample collection, ribonucleic acid (RNA) extraction, complementary deoxyribonucleic acid (cDNA) synthesis, RT-qPCR, RT-cPCR, multiplex tiling PCR, Oxford Nanopore sequencing, consensus genome generation, and phylogenetic analyses (Lines 141-272). |
| Bias | | 9 | Describe any efforts to address potential sources of bias:  Potential sources of bias are discussed in the Study Limitations section, including the limited number of sequenced samples, incomplete genomic coverage, loss of amplicons, platform-specific biases, and limited geographical sampling (Lines 451-486). |
| Study size | | 10 | Explain how the study size was arrived at:  The study size was determined by the number of animals sampled during the cross-sectional survey. No formal power analysis was performed because this was an exploratory genomic investigation. The number of genomes available for analysis was further limited by RT-qPCR positivity and successful recovery of near-whole-genome sequencing. |
| Quantitative variables | | 11 | Explain how quantitative variables were handled in the analyses. If applicable, describe which groupings were chosen and why:  Quantitative variables, including RT-qPCR Cq values, sequencing depth, genome coverage, nucleotide identity, and phylogenetic support value, are described together with their analytical use. |
| Statistical methods | | 12 | (*a*) Describe all statistical methods, including those used to control for confounding:  Descriptive statistics were used to summarise sample characteristics and PPRV detection. Phylogenetic analyses were performed using maximum-likelihood methods, and pairwise nucleotide identities were calculated. No multivariable statistical models were required. |
|  |  |  | (*b*) Describe any methods used to examine subgroups and interactions:  Not applicable. No subgroup or interaction analyses were performed. |
|  |  |  | (*c*) Explain how missing data were addressed:  Samples failing RT-cPCR confirmation or multiplex PRC amplification were excluded from genome sequencing. Genome coverage gaps resulting from amplicons dropout are described in the Results and Study Limitations sections. |
|  |  |  | (*d*) *Cohort study*-If applicable, explain how loss to follow-up was addressed  *Case-control study*-If applicable, explain how matching of cases and controls was addressed  *Cross-sectional study*-If applicable, describe analytical methods taking account of sampling strategy:  Not applicable. This was a cross-sectional study without follow-up. The analytical approach did not require adjustment for sampling strategy. |
|  |  |  | (*e*) Describe any sensitivity analyses:  Not applicable. No sensitivity analyses were performed due to the exploratory nature of the genomic investigation and the limited number of successfully sequenced genomes. |
| Results | | | |
| Participants | 13* | (a) Report numbers of individuals at each stage of study-eg numbers potentially eligible, examined for eligibility, confirmed eligible, included in the study, completing follow-up, and analysed:  The numbers of sampled animals (n = 355), RT-qPCR-positive samples (n = 25), RT-cPCR-positive samples (n = 17), samples selected for sequencing evaluation (n = 10), and successfully sequenced genomes (n = 4) are reported in the Results section (Lines 299-320). | |
|  |  | (b) Give reasons for non-participation at each stage:  Not applicable. However, reasons for exclusion from genome sequencing (failure of RT-cPCR confirmation, poor multiplex PCR amplification, or insufficient sequencing quality) are described in the Results section (314-320). | |
|  |  | (c) Consider use of a flow diagram:  Not applicable. | |
| Descriptive data | 14* | (a) Give characteristics of study participants (eg demographic, clinical, social) and information on exposures and potential confounders:  Characteristics of the sampled animals, including species, breed, age, sex, and geographical distribution, are presented in the Results section (Lines 299-320) and Table 1 (Line 769). | |
|  |  | (b) Indicate number of participants with missing data for each variable of interest:  There were no missing demographic data. Samples failing laboratory quality criteria were excluded from sequencing and are described in the Results section and Table 1. | |
|  |  | (c) *Cohort study*-Summarise follow-up time (eg, average and total amount):  Not applicable. | |
| Outcome data | 15* | *Cohort study*-Report numbers of outcome events or summary measures over time | |
|  |  | *Case-control study-*Report numbers in each exposure category, or summary measures of exposure | |
|  |  | *Cross-sectional study-*Report numbers of outcome events or summary measures:  The numbers of RT-qPCR-positive samples, RT-cPCR-positive samples, successfully sequenced genomes, genome coverage metrics, pairwise nucleotide identity values, and phylogenetic finding are reported in the Results section (Lines 297-352). | |
| Main results | 16 | (*a*) Give unadjusted estimates and, if applicable, confounder-adjusted estimates and their precision (eg, 95% confidence interval). Make clear which confounders were adjusted for and why they were included:  Not applicable. | |
|  |  | (*b*) Report category boundaries when continuous variables were categorized:  Not applicable. | |
|  |  | (*c*) If relevant, consider translating estimates of relative risk into absolute risk for a meaningful time period:  Not applicable. | |
| Other analyses | 17 | Report other analyses done-eg analyses of subgroups and interactions, and sensitivity analyses:  Not applicable. | |
| Discussion | | | |
| Key results | 18 | Summarise key results with reference to study objectives:  The Discussion summarises the principal findings with reference to the study objectives (Lines 353-449). | |
| Limitations | 19 | Discuss limitations of the study, taking into account sources of potential bias or imprecision. Discuss both direction and magnitude of any potential bias:  The limitations of the study, including limited sample size, incomplete genome coverage, Oxford Nanopore-specific sequencing biases, and restricted geographical coverage, are discussed in detail (Lines 451-486). | |
| Interpretation | 20 | Give a cautious overall interpretation of results considering objectives, limitations, multiplicity of analyses, results from similar studies, and other relevant evidence:  The Discussion provides a balanced interpretation of the findings in the context of previous studies while acknowledging the study limitations. | |
| Generalisability | 21 | Discuss the generalisability (external validity) of the study results:  The Discussion notes that the findings specifically reflect PPRV circulation in northern Cote d'Ivoire and may not be representative of the entire country. The broader regional context is discussed by comparison with lineage IV circulation in West Africa. | |
| Other information | | | |
| Funding | 22 | Give the source of funding and the role of the funders for the present study and, if applicable, for the original study on which the present article is based:  The Funding section identifies the funding source (PASET-RSIF, Project Grant No. P165581) and states that the funders had no role in the study design, data collection, analysis, interpretation, manuscript preparation, or publication decision (Lines 537-544). | |

*Give information separately for cases and controls in case-control studies and, if applicable, for exposed and unexposed groups in cohort and cross-sectional studies.

**Note:** An Explanation and Elaboration article discusses each checklist item and gives methodological background and published examples of transparent reporting. The STROBE checklist is best used in conjunction with this article (freely available on the Web sites of PLoS Medicine at http://www.plosmedicine.org/, Annals of Internal Medicine at http://www.annals.org/, and Epidemiology at http://www.epidem.com/). Information on the STROBE Initiative is available at www.strobe-statement.org.
